# Application of bundled process control in the prevention of pressure injury in patients with head and neck cancer

**DOI:** 10.1371/journal.pone.0305190

**Published:** 2024-06-10

**Authors:** Mianmian Chen, Fenfen Wang, Xueying Xie, Xiaohong Yang, Yaling Luo, Chaoman Zhuang, Baoyuan Xie

**Affiliations:** 1 Department of Otolaryngology, The Second Affiliated Hospital of Fujian Medical University, Quanzhou, Fujian Province, China; 2 Department of Nursing, The Second Affiliated Hospital of Fujian Medical University, Quanzhou, Fujian Province, China; National Trauma Research Institute, AUSTRALIA

## Abstract

This study aimed to explore the application effects of cluster process control and routine nursing on the prevention of pressure injury (PI) in patients undergoing head and neck cancer surgery and to provide a basis for reducing the occurrence of PI, thereby promoting the safety of the patients. This was a retrospective study. Patients with head and neck cancers who underwent surgical treatment in the Department of Otolaryngology at the Second Affiliated Hospital of Fujian Medical University from July 2022 to June 2023 were selected as the research participants. Participants were classified into experimental and control groups using a convenience sampling method. In the experimental group, cluster process control was implemented, while routine nursing management was applied in the control group. The incidence of PI (*p* = 0.028) and healing time (*p* = 0.035) in the experimental group were lower than those in the control group. The process management ability of nurses in the experimental group was significantly improved, with the results for the Braden scale (*p* = 0.023), effective decompression (*p* = 0.002), floating heel (*p* = 0.002), nutrition monitoring (*p* = 0.005), and patient satisfaction in the experimental group being higher than those in the control group (*p* = 0.007). This study effectively demonstrated the effect of cluster process control in reducing the incidence of PI in patients undergoing head and neck cancer surgery, thereby determining that cluster process control is suitable for clinical application.

## Introduction

Head and neck cancer is the seventh most common cancer globally, accounting for more than 660,000 new cases annually [1]. In addition to receiving radiotherapy to treat cancers such as nasopharyngeal carcinoma, many patients with head and neck cancer undergo surgical treatment. Due to the long time spent in the supine position during the operation, followed by the long time spent in bed afterward, the incidence of pressure injury (PI) among patients with head and neck cancer is high. A study has shown that in patients undergoing long-term head and neck cancer resection and reconstruction surgery, PI usually occurs in the bone protuberance. Increased operation time has also been proven to be a statistically significant factor in the occurrence of PI in this group of patients [2].

The 2019 European Pressure Ulcer Advisory Panel (EPUAP)/National Pressure Injury Advisory Panel (NPIAP)/Pan Pacific Pressure Injury Alliance (PPPIA) guideline redefined PI as a localized injury that occurs in the skin or subcutaneous soft tissue [3]. PI usually occurs at the bone protuberance, but it may be associated with medical devices or other similar items. Pressure sores can cause pain in patients, significantly increase nursing time and hospitalization costs, affect rehabilitation and delayed discharge, and lead to death [4]. The number of patients with PI in the United States is as high as 6.5 million annually, and the cost of treatment is about 25 billion USD [5]. In the UK, pressure ulcer treatment costs account for approximately 4% of public healthcare expenditure, with nursing time accounting for 41% of these costs [6].

In 2012, the Institute for Healthcare Improvement (IHI) and the National Health Service (NHS) recommended the Skin, Surface, Keep moving, Incontinence, and Nutrition (SSKIN) cluster prevention as the best practice [7, 8]. Kennedy [9] reported that the SSKIN cluster nursing program had a definite effect on preventing PI, but to date, there has been no study that addressed this issue and made clear recommendations for patients who have undergone head and neck cancer surgery. Therefore, the purpose of this study was to extensively assess the potential preventive effect of PI cluster process control on the occurrence of PI in patients who have undergone head and neck cancer surgery. Furthermore, we aimed to construct a set of nursing norms that focus on PI prevention in patients who have undergone surgery.

## Methods

### Patient characteristics

This retrospective study was conducted in September 2023 and involved patients with head and neck cancer who were admitted to the otolaryngology department of our hospital from July 2022 to the end of June 2023. A convenience sampling method was used to classify participants into the experimental and control groups. The experimental group was managed using cluster process control, while routine nursing management was applied in the control group. Patient data were collected from inpatient medical records, information registration forms, checklists, and satisfaction questionnaires. The information registration form included details on sex, age, length of hospital stay, duration of surgery, disease diagnosis, body mass index (BMI), preoperative nutrition score, and Braden score of the patients. The checklist included details on whether the patient had a pressure injury, wound care and healing, and the implementation of nursing measures. The inclusion criteria were a formal diagnosis of head and neck cancer confirmed by pathology and undergoing surgical treatment; an expected operation time of >4 hours and a hospitalization time >7 days; age ≥18 years; and a provision of informed consent by patients or their families. The exclusion criteria were a diagnosis of skin diseases that affected skin observation; occurrence of PI before admission; diagnosis of severe cardiovascular, cerebrovascular, or endocrine diseases; canceled surgery or an unplanned secondary surgery within 72 hours; and active withdrawal from the study or inability to complete the study due to other reasons (Fig A in S1 File). Patients diagnosed with head and neck cancer who met the inclusion criteria from June 2022–December 2022 (before the implementation of the SSKIN cluster process control) were included in the control group. From January 2023–June 2023, patients who met the inclusion criteria and underwent head and neck cancer surgery (after the implementation of SSKIN cluster process control) were included in the experimental group. The Braden scale was used for the PI risk assessment [10], and the staging criteria were the 2019 EPUAP/NPIAP/PPPIA guidelines [3]. Nutritional Risk Screening 2022 (NRS2002) was used as a nutritional risk assessment scale [11].

This study was conducted according to the Helsinki Declaration and the Clinical Practice Guidelines and approved by the Ethics Review Committee of the Second Affiliated Hospital of Fujian Medical University (No. 411, 2023). Written informed consent was obtained from the patients. All procedures in studies involving human participants were performed in accordance with the ethical standards of the Institutional Research Council, and all methods were conducted in accordance with the relevant guidelines and regulations.

### Intervention methods

The control group received routine nursing management: skin examinations were performed at admission, before and after surgery, and at each change of position. The protocol also involved the provision of nursing measures by the responsible nurse to ensuring that the patient’s skin was cleaned on time, the patient’s clothing and bed unit were kept dry and comfortable. One day before and after surgery, the skin at the bone process was evaluated, the abnormalities were recorded, and local protection was provided to high-risk patients if necessary, for example, by the addition of foam dressing to protect the bone protrusion site. Patients were turned over in the bed once every 2 hours. Decompression and body position mattresses were used before and after the operation. Structured skin care was applied to moist skin, PI wounds were treated with wet dressings, and ostomy and wound therapist consultation guidance were requested if necessary. Routine nutritional assessment was also implemented, with nutritional intervention provided according to a doctor’s advice. The head nurse periodically organized the discussion and analysis of the cases of pressure injury. The quality control group leader checked the nursing quality once a quarter.

In accordance with routine nursing practices, an evidence-based analysis was performed for the experimental group after summarizing nursing measures and the adopted SSKIN clustering process control. A PI cluster nursing team for head and neck cancers was established, led by the head nurse of the relevant department. One enterostomal therapist (ET), one wound specialist team nurse, six backbone nurses specializing in otolaryngology, and one registered dietitian participated in patient management. The education plan was formulated, training was unified, and the division of labor was clear.

Skin assessment: According to the content and objectives of the PI assessment, the ET trained nurses with teaching demonstrations and real-life operation methods to enable them to acquire knowledge and improve their PI assessment ability. Visual, palpation, and inquisition techniques were used to assess the patient’s whole-body skin condition upon admission. The skin conditions of sacrococcygeal, scapular, elbow, ankle, heel, and other bone processes were evaluated and recorded 3 days prior to the patient’s scheduled operation. One day before the operation, the skin condition of the bone process was evaluated, and data were recorded and photographed. After the operation, the patient’s skin was examined from head to toe, and pain at the bone process was evaluated and recorded. Any abnormal skin region was photographed for accurate data retention. After each turn and handover, the skin over the bone processes, the prevention dressing, and the patient’s level of pain were evaluated and recorded. Under the premise of ensuring safe use, medical devices were removed once to twice each day, and the condition of the skin under the medical device was evaluated and recorded. The ET checked the application of risk assessment weekly, and the head nurse supervised the assessment process and training every month; this was to ensure that the nurses mastered the comprehensive and dynamic risk assessment and improved in their screening and assessment abilities.

**Fig 1:**
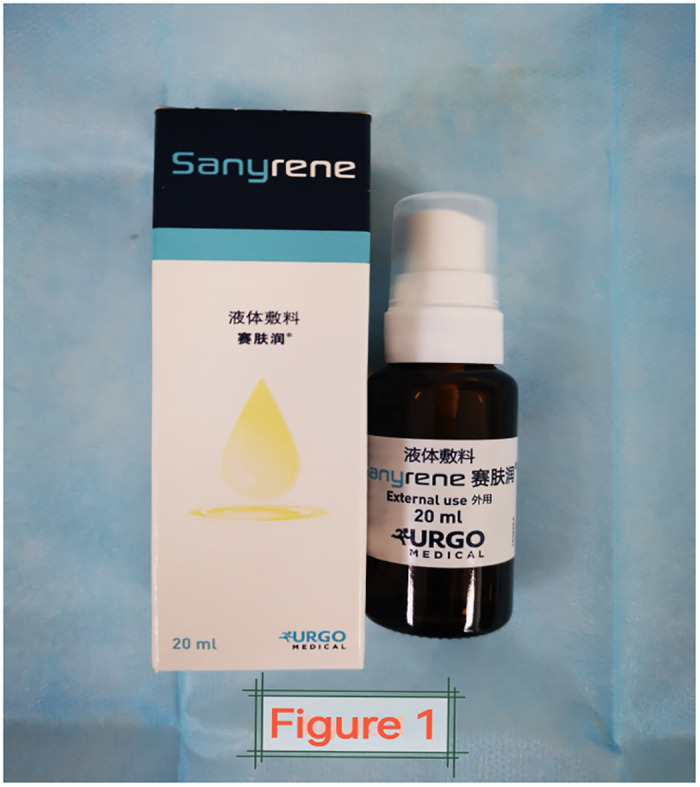
Oily liquid dressing to prevent PI. Sanyrene, applied two to three times a day. Sanyrene is sprayed on the complete skin of the sacrococcygeal, scapular, elbow, ankle, heel, and other bone processes in patients with head and neck cancers and applied evenly.

**Fig 2:**
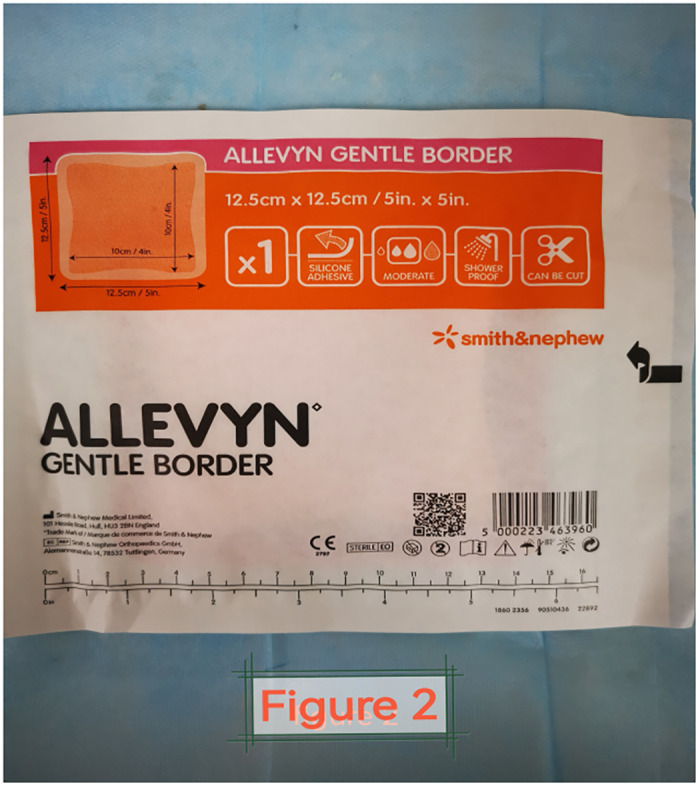
Wound dressing to prevent PI. Silicone adhesive foam dressing is used to protect PI-high-risk skin and PI wound care in patients with head and neck cancers. After Sanyrene is sprayed and absorbed, it is pasted on the sacrococcygeal, scapular, elbow, ankle, heel, and other bone processes of the patient and replaced every 1 to 2 days. For PI wound care, the amount of exudation is evaluated to determine the frequency with which replacement is required. PI, pressure injury.

Skin’s surface: Three days before surgery, the patient’s skin at the sacrococcygeal, scapular, elbow, ankle, and heel was sprayed with liquid dressing (Fig 1) twice per day. Before entering the operating room, liquid dressings were sprayed on the skin at the bone processes, and silicone foam dressings were pasted after absorption (Fig 2). A decompression mattress was used in the operating room. Before the operation, the foam dressing at the bone processes was examined to ensure that its positioning was flat without any risk of loosening or displacement. The gel pad was used for decompression at the main compression site. After the operation, liquid and foam dressings were used to protect the skin at the bone processes, and the soft pad was placed under the gastrocnemius muscle to properly float the heel for decompression. The head nurse regularly reviewed the use of preventive dressings and the position management of high-risk patients to evaluate the correctness and effectiveness of the preventive measures taken.

Regular turning of the patients: The body compression points of the supine and lateral positions were marked in the form of a map, and the best position for placing the position pad in different supine positions was correctly demonstrated. The frequency of turning over was formulated according to the patient’s individual activity level and independent position-changing ability. The decompression mattress and nursing skills were used to assist the patient in changing their supine position, and the bedside elevation angle was below 30°. The ET and wound team members used bedside teaching methods to guide the nurses to accurately judge whether the decompression method was effective after turning over. The head nurse organized the scenario simulation. The nurses took turns playing bedridden patients, and other nurses assisted in the turning over. The nurses who acted as bedridden patients described the feelings they experienced in terms of body compression while turning over and decompression after turning over so that all nurses could master their turning-over skills.

Incontinence: The PI learning package was compiled, and the PI staging was presented in color maps and provided as part of the treatment plan, making it convenient for the nurses to look at for comparison at any time. Skin moisture caused by incontinence, sweat, or exudation was managed, preventive dressings were changed or removed daily, local skin hydration levels were assessed, and skin health with a structured care process of skin cleaning, moisturizing, and skin care was maintained. Wet wound dressings were selected to maintain humidity balance or reduce humidity as required. ET or wound team members monitored the healing process of the PI, continuously tracked the physiological characteristics of the wound bed and the surrounding skin and soft tissue, and evaluated the nursing effect.

Nutrition: Patients with preoperative nutritional risk or those who were aged ≥ 70 years were provided with nutritional intervention after admission, providing 1.25–1.5 g/kg/d body weight protein and 30–35 kcal/kg/d body weight calories. On the first day after the operation, patients were administered a nasal feeding liquid diet to maintain enteral nutrition intervention without contraindications. Nurses on each shift were required to assess whether the patient’s nutritional intake was sufficient and monitor their excretion status, while nutrition-related indicators were monitored weekly. These indicators included protein level, hemoglobin, arm circumference, and triceps skinfold thickness. This enabled the correct assessment of the nutritional status of patients, alongside discussion with doctors, patients, and caregivers to develop a nutritional care plan. Patients who exhibited changes in their condition, reduced food intake, or fasted for more than 3 days were asked to consult a registered dietitian to determine the patient’s requirements for heat, protein, and liquid. This enabled a personalized nutrition intervention program to be developed to improve the overall nutritional supply and improve skin tolerance.

The occurrence of PI, PI healing time, nurses’ process management ability, and patient satisfaction were observed and recorded in both the experimental and control groups. The incidence of PI was divided into stages 1, 2, 3, 4, deep tissue injury, and unstaged according to the EPUAP/NPIAP/PPPIA guidelines published in 2019 [3]. The number of PI cases in the two groups within 72 hours post-operation was recorded.

The incidence of PI = (the number of patients with PI in the cycle/the number of cases in this group) × 100%.

The number of patients with multiple PIs (n >1) in the two groups was also recorded.

The incidence of multiple PIs = (the number of patients with multiple PIs in the cycle/the number of patients with PI in this group) × 100%.

PI median healing time: The PI healing time of the two groups was recorded, and the median was calculated.

Correct rate of nurse process management: Correct scoring of the Braden scale, effective turn-over decompression rates, heel floating rates, and nutritional support rate of the two groups were recorded.

Correct rate = (the number of patients who correctly implemented process management in the cycle/the number of cases in this group) × 100%.

This self-made scale was used to evaluate nursing communication, attitude, operation, and communication flow 1 week post-operation. The scores were classified as satisfied, basically satisfied, and not satisfied (Table A in S1 File).

### Statistical methods

An Excel table was used for data entry, and the SPSS 26.0 statistical software package was used for data analyses. All data were tested for normality. The count data are expressed as the number of cases and the corresponding percentage rate (%). The comparison between the two groups was performed using the chi-squared test. Measurement data with a normal distribution are statistically described as mean ± standard deviation $\left(\bar{\text{x}}\pm \text{s}\right)$, and an independent sample t-test was used for comparison between groups. Measurement data that were not normally distributed were statistically described as median (interquartile range), and a rank sum test was used for comparison between groups; *p*<0.05 was considered statistically significant.

## Findings

### Comparative analysis of general data of research participants

A total of 127 patients with head and neck cancers who underwent surgical treatment in the Department of Otolaryngology at the Second Affiliated Hospital of Fujian Medical University from July 2022–June 2023 were selected as the research participants in this study. The study sample included 101 males and 26 females and had an average age of 60.18±11.65 years. The control group comprised 60 patients who underwent head and neck cancer surgery with routine care, while the experimental group comprised 67 patients who underwent head and neck cancer surgery after SSKIN cluster process control. There was no significant difference in the general demographic data between the two groups (*p*>0.05), indicating that the data of the two groups were comparable, as shown in Table 1.

**Table 1:** Comparative analysis of the general data of the two groups of patients.

Item	Category	Experimental group (n = 67)	Control group (n = 60)	χ^2^/t	p-value
Sex, n	Females	14(20.90%)	12(20.00%)	0.109	0.741
	Males	53(79.10%)	48(80.00%)		
Age, yr (mean±SD)		59.99±10.95	60.37±12.34	-0.185	0.854
Hospital days (d)		20.69±7.28	20.97±6.44	-0.228	0.820
Operation time (h)		8.45±2.99	9.39±2.76	-1.835	0.069
NRS2002 scale		1.55±0.70	1.62±0.76	-1.485	0.140
BMI		23.00±2.61	23.60±3.17	-1.173	0.201
Braden scale		22.70±0.46	22.77±0.43	-0.824	0.412
Disease diagnosis (n)	Carcinoma of floor of mouth	5(7.46%)	3(5.00%)	1.768	0.940
	Cheek cancer	6(8.96%)	6(10.00%)		
	Gingival cancer	4(5.97%)	5(8.33%)		
	Hypopharyngeal carcinoma	9(13.44%)	8(13.33%)		
	Laryngeal cancer	18(26.86%)	12(20.00%)		
	Tongue cancer	18(26.86%)	17(28.34%)		
	Other	7(10.45%)	9(15.00%)		

### Incidence of PI, median healing time, process management, and satisfaction

Among the 67 patients in the experimental group, 12 (17.91%) had PI, of which 5 (41.67%) had multiple PIs. In the control group, there were 21 cases (35.00%) of PI among the 60 patients, including 10 (47.62%) with multiple PIs. There was a significant difference in the incidence of PI between both groups (χ^2^ = 4.807, *p* = 0.028). The median healing time of PI was statistically significant (U = 70.500, Z = -2.109, *p* = 0.035). The nurse process management protocol included: correct assessment of the Braden scale (χ^2^ = 5.190, *p* = 0.023), correct and effective decompression (χ^2^ = 9.904, *p* = 0.002), correct floating heel (χ^2^ = 9.147, *p* = 0.002), correct nutrition monitoring (χ^2^ = 7.961, *p* = 0.005), with the differences being statistically significant. There was also a statistically significant difference in the level of patient satisfaction (χ^2^ = 7.358, *p* = 0.007), as shown in Table 2. There was no significant difference in the incidence of multiple PI between the two groups (χ^2^ = 0.109, *p* = 0.741), as shown in Table 3.

**Table 2:** Incidence of PI, healing time, process management, and satisfaction in the two groups.

Item	Experimental group (n = 67)	Control group (n = 60)	χ^2^/t	p-value
PI(n)				
Yes	12(17.91%)	21(35.00%)	4.807	0.028*
No	55(82.09%)	39(65.00%)		
Median healing time(d)				
	2(95%CI = 1.16~4.00)	3(95%CI = 2.86~5.14)	-2.109	0.035*
Correct process management(n)				
Assessing the Braden scale	58(86.57%)	42(70.00%)	5.190	0.023*
Effective decompression	60(89.55%)	40(66.67%)	9.904	0.002*
Floating heels	61(91.04%)	42(70.00%)	9.147	0.002*
Nutrition monitoring	66(98.51%)	51(85.00%)	7.961	0.005*
Satisfaction situation(n)				
Satisfied	32(47.76%)	19(31.67%)	7.358	0.007*
Basically satisfied	27(40.30%)	22(36.66%)		
Not satisfied	8(11.94%)	19(31.67%)		

* p<0.05, have statistical significance.

**Table 3:** Analysis of the incidence of multiple PI in the two groups of patients.

Item	Experimental group (n = 12)	Control group (n = 21)	χ^2^	p-value
Multiple PI(n)				
Yes	5(41.67%)	10(47.62%)	0.109	0.741
No	7(58.33%)	11(52.38%)		
PI staging(n)				
Stage 1	7(58.34%)	12(57.14%)	1.928	0.381
Stage 2	1(8.33%)	0		
Stage 3	0	0		
Stage 4	0	0		
Unstageable	0	0		
Deep Tissue	4(33.33%)	9(42.86%)		

## Discussion

### Relationship between PI and surgical patients with head and neck cancers

**Fig 3:**
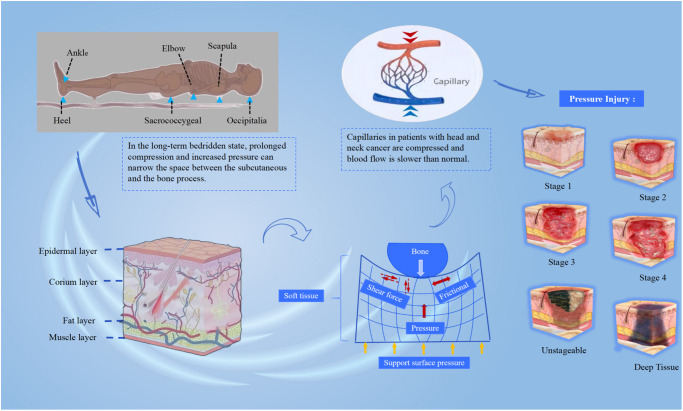
The mechanism of PI in patients with head and neck cancer post-operation. Patients with head and neck cancers are prone to compression in the pillow, scapula, sacrococcygeal, heel, and elbow when they are placed in the supine position for a lengthy period of time. PI is defined as the continuous deformation of local skin or subcutaneous tissue caused by high or long-term pressure or shear stress, which distorts the capillary network and reduces the nutritional supply to the tissue. The injury usually occurs at the bone process, which can manifest as a complete skin or open wound and may be accompanied by pain. The NPIAP system is commonly used in clinical practice to classify PI according to the following stages: Stage 1 PI, Stage 2 PI, Stage 3 PI, Stage 4 PI, Unstageable PI, and Deep Tissue PI. NPIAP, National Pressure Injury Advisory Panel; PI, pressure injury.

Surgical patients have a high risk of developing PI [12, 13]. Pressure is the most important contributing factor in the occurrence of PI, and it is difficult to achieve zero occurrence. The incidence of PI is one of the quality monitoring indicators for evaluating patient care safety. The results of Tura et al. [14] and Kozhimala et al. [15] showed that the duration of surgery is a contributing factor for PI, with a procedural duration greater than 4 hours being a significant predictor of postoperative skin injury. Patients with head and neck cancers are placed in a supine position for a long time during surgery, exacerbating the challenges faced with regard to their own basic diseases and the risks posed by long-term skin compression. These patients are thus prone to experiencing compression at the point of the pillow, scapula, sacrococcygeal bone, heel, and elbow. Ceylan et al. investigated 46 patients in the supine position and found that the blood oxygen of sacrococcygeal tissue increased with the extension of compression time, then decreased, and then remained stable [16]. Peirce monitored the effects of local compression of the skin in rats, with the results obtained showing that blood flow was decreased by 80% after 2 hours of compression [17]. Continuous vertical pressure and shear stress cause tissue deformation and cell damage (Fig 3). The mean operation time in PI patients in this study was 10.69±2.34 h, and that in patients without PI was 8.26±2.84 h. The difference observed was statistically significant (t = 4.419, *p*<0.001), indicating that the occurrence of PI in this study was closely related to long-term compression, which was consistent with the results of previous studies [14, 15].

### Effect of bundled process control on PI in patients undergoing head and neck cancer surgery

A comprehensive, objective, and accurate assessment of PI risk is the premise of the correct selection of preventive measures. According to the results of risk assessment, the prevention and management plan can reduce the interference of risk factors and reduce the occurrence of pressure injury. The 2016 Wound, Ostomy, and Continence Nurses Society (WOCN) guidelines [18] recommend that patients at high risk of PI consider using preventive dressings to protect the sacrococcygeal and heel bones and minimize the pressure and shear force placed on both the body and the support or contact surface between the body and the medical device to reduce the risk of PI [19]. The effect of foam dressing on preventing PI was previously confirmed in a randomized controlled trial [20]. Another study by Yoshimura et al. has shown that soft silicone foam dressings have better preventive effects than polyurethane coating dressings in patients undergoing spinal surgery in a prone position [21]. Liquid dressings can increase local skin water content, improve skin elasticity, and prevent pressure, shear force, and friction from damaging the skin or tissue [22]. The use of an appropriate support surface during the operation can also effectively reduce the occurrence of PI [23]. Moisture-related skin damage can damage the epidermal barrier function and easily lead to tissue PI [24]. Malnutrition also increases the risk of PI. A common clinical problem occurs when nurses only take note of problematic nutrition scores but do not participate in the follow-up intervention [25].

In this study, nurses’ risk assessment ability was improved through teaching demonstrations and real-life operation training, and the assessment process was formulated to provide evidence for nurses. Nurses were guided to pay attention to the state of the patient’s skin surface and choose the correct preventive dressings to reduce the pressure on the skin and the resulting damage. Effective turn-over decompression is the key prevention link. Being aware of this fact allows nurses to feel the pressure of bed rest and the comfort of turn-over decompression, allowing them to implement individualized position management and establish a turn-over timetable to provide effective turn-over decompression for patients. Using structured skin care to avoid skin exposure to excessive humidity, protect dry skin, and promote hydration can help prevent skin damage. An important measure for preventing PI is for nurses to dynamically evaluate patient food intake and digestion and absorption capacity, as well as improve the nutritional status of patients under the professional guidance of nutritionists. In this study, SSKIN cluster measures were adopted, and the incidence of PI decreased from 35.00% before implementation to 17.91% after implementation. The difference was statistically significant (χ^2^ = 4.807, *p* = 0.028), indicating that the cluster process control effectively reduced the incidence of PI in patients undergoing head and neck cancer surgery, a finding that is consistent with previous research results [9, 26]. In this study, two groups of patients underwent surgery in a supine position for a lengthy period. The location of the PI in the experimental group was sacrococcygeal > elbow > ankle > scapula > heel. The incidence of multiple PI in the control group was sacrococcygeal > elbow > scapula > heel = ankle (Table B and Fig B in S1 File); there was no significant difference in the incidence of multiple PI between the two groups (χ^2^ = 1.109, *p* = 0.741). In both groups, stage 1 PI was more common, followed by deep tissue injury, and the difference was not statistically significant (χ^2^ = 1.928, *p* = 0.381).

The variables that affected the PI healing time included PI staging, initial area, microcirculation, nutritional status, and rationality and adequacy of measures [27, 28]. In this study, the knowledge training provided around injury identification, skin protection, and wound treatment was carried out using various media, such as teaching videos, scenario simulations, case discussions, and learning packages. Together, these measures helped improve the wound treatment abilities of the nurses who partook in this study. Stage 1 PI with local invariant white erythema was found to be most common in this study, followed by deep tissue damage with dark red and purple skin integrity. In the bundled process control, the stoma therapist and wound care team continued to track the wound treatment process and achieved positive results through effective nursing. The PI median healing time in the experimental group was lower than that in the control group, with the difference being statistically significant (Z = -2.109, *p* = 0.035). The average cost of wound care in the experimental group was ¥681.46±231.20, which was lower than that of the control group (¥803.50±258.10), however, the difference was not statistically significant (t = -1.355, *p* = 0.185). Halim et al. have shown that wound bed preparation and clinical management can achieve a balanced, moist wound, accelerate healing, and correct biochemical imbalances [29]. Allen also showed that the nutritional regimen effectively improved PI wound healing [30]. In this study, the PI wound was treated according to the theory of wet balance, and the implementation of nutritional intervention in the prevention and management of PI was emphasized. The results obtained further confirmed the accuracy of these views. The occurrence and development of PI is a gradual process, and preventive measures should be taken throughout the perioperative period. The head nurse’s executive leadership function involves promoting the construction of the skin management nursing team [31], centralizing management and coordination, and regularly evaluating the implementation and preventive effects. Leadership and communication skills are very important; frequent communication with the team is crucial for implementing a new process [9]. The clinical leadership program is considered part of a successful quality improvement program, including the appointment of personnel who have obtained professional qualifications through certification project training to provide prevention and treatment of stress injuries as part of the quality improvement process [32, 33]. ETs play an extremely important role in this professional field. Through continuous training and collaboration, a skincare team was formed to ensure that each nurse can undertake PI management in each nursing position and link. After the comparative analysis of the two groups in this study, statistically significant differences were found in the process control of the nurses in all aspects, including the correct evaluation of the Braden scale (χ^2^ = 5.190, *p* = 0.023), correct and effective decompression (χ^2^ = 9.904, *p* = 0.002), correct floating heel (χ^2^ = 9.147, *p* = 0.002), and proper nutrition monitoring (χ^2^ = 7.961, *p* = 0.005). This shows that the implementation of SSKIN clustering process control can effectively improve the overall implementation ability of nurses. Related research results have shown that single intervention measures (preventive dressing, support surface, reduction, preventive skin care, system reminder, and medical staff education) are effective in reducing PI [34] and that multiple intervention programs are more effective than single intervention programs in reducing the occurrence of PI. At least 12 body position changes per day are required to be considered as frequent turn-overs, and frequent turn-overs can reduce the incidence of stress injuries in high-risk patients [35]. In this study, as per the implementation of the SSKIN cluster process control, nurses increased the time allocation for patient position management. The average nursing time for body position management in the experimental group was 31.61±4.12 min, which was higher than that in the control group (28.60±6.28 min), and the difference was statistically significant (Z = -2.805, *p* = 0.005). Factors such as staffing characteristics, knowledge and attitudes, appropriate equipment, and resources are the main issues that can promote or hinder practice [3, 36]. This study evaluated the environment for implementing the plan and introduced available advantageous factors before developing a quality improvement plan. The results have confirmed that cluster process control can improve the effectiveness of nursing engagement in multiple interventions to reduce the incidence of PI. After the implementation of the SSKIN cluster process control in patients with head and neck cancer, the comparative analysis of the two groups revealed that patient satisfaction was significantly improved, and the difference was statistically significant (χ^2^ = 7.358, *p* = 0.007). Strong scores were recorded relating to nursing communication, attitude, operation, and notification options, indicating that the medical care experience of patients in the experimental group was significantly improved.

The respiration, swallowing, and eating functions in patients with head and neck cancers are affected because of the particularity of the relevant tumor locations; this, in turn, affects these patients’ quality of life. Therefore, postoperative nursing is highly important in this treatment area. SSKIN cluster process control effectively improves the work practice and management efficiency of nurses at all levels, tracks perioperative skin care measures, improves outcomes regarding postoperative complications, improves the quality of clinical care, and improves patient satisfaction. Considering the specific needs of patients with head and neck cancers, attempts must be made to continue to improve this mode of nursing and further improve the postoperative recovery efficiency for these patients.

This study has several limitations. This was a retrospective study, which means that the sampling strategy may be biased. Additionally, this was a single-center study with a small sample size. In the future, multi-center studies will be carried out to expand the sample size for further prospective studies and improve the promotion and implementation of the program.

## Conclusions

SSKIN cluster nursing is a crucial part of the PI prevention principle. In this study, cluster process control was used to reduce the incidence of PI in patients undergoing head and neck cancer surgery, shorten the median healing time, and improve the process management ability of nurses and overall patient satisfaction. The results obtained have strong applicability and practicality in clinical nursing settings, with the aim of continuous quality improvement.

## Supporting information

S1 File (DOCX)
