# Supplementary material for: Application of bundled process control in the prevention of pressure injury in patients with head and neck cancer
Source: PLoS One. 2024 Jun 10;19(6):e0305190. doi: 10.1371/journal.pone.0305190 (PMC11164361; doi:10.1371/journal.pone.0305190)
Supplement: S1 File — (DOCX) [file pone.0305190.s001.docx]

**Satisfaction Questionnaire for Nursing Work and Nursing Effect**

**Dear Patient:**

Thank you for choosing the Department of Otolaryngology at the Second Affiliated Hospital of Fujian Medical University. Our team provided medical services for you. This is a questionnaire about your satisfaction with nursing work and nursing effects during treatment, including four aspects: nursing communication, attitude, operation, and communication. The scores are classified as satisfied, basically satisfied, and not satisfied.

1. Satisfied: The nursing work and nursing effects exceed or fully meet your expectations; the nurse's performance is very good.
2. Basically satisfied: The nursing work and nursing effect meet your expectations but can do better.
3. Not satisfied: There is a gap between nursing work and nursing effects and your expectations, and the quality of nursing needs to be improved.

Please use “√” to represent your degree of satisfaction in the following table. We thank you for your participation and wish you a speedy recovery.

| **Table A. Satisfaction questionnaire of nursing work and nursing effect** | | | |
| --- | --- | --- | --- |
| Item | Satisfied | Basically satisfied | Not satisfied |
| Communication |  |  |  |
| Attitude |  |  |  |
| Operation |  |  |  |
| Communication |  |  |  |

**Table B.** **Analysis of the Location of Multiple PIs in the Two Groups of Patients.**

| Item | Experimental group  （n=19) | Control group  (n=33) | χ² | p-value |
| --- | --- | --- | --- | --- |
| Location of Multiple PI(n) | | | | |
| Sacrococcygeal | 8(42.1%) | 13(39.4%) | 1.201 | 0.878 |
| Elbow | 5(26.3%) | 8(24.2%) |  |  |
| Ankle | 3(15.8%) | 3(9.1%) |  |  |
| Scapula | 2(10.5%) | 6(18.2%) |  |  |
| Heel | 1(5.3%) | 3(9.1%) |  |  |

Figure A: Flow Chart of Patients’ Inclusion and Exclusion Criteria

Figure B: Histogram of Multiple PIs Location in the two groups of patients
